# Influenza D Virus Infection in Feral Swine Populations, United States

**DOI:** 10.3201/eid2406.172102

**Published:** 2018-06

**Authors:** Lucas Ferguson, Kaijian Luo, Alicia K. Olivier, Fred L. Cunningham, Sherry Blackmon, Katie Hanson-Dorr, Hailiang Sun, John Baroch, Mark W. Lutman, Bianca Quade, William Epperson, Richard Webby, Thomas J. DeLiberto, Xiu-Feng Wan

**Affiliations:** Mississippi State University, Starkville, Mississippi, USA (L. Ferguson, K. Luo, A.K. Olivier, S. Blackmon, H. Sun, B. Quade, W. Epperson, X.-F. Wan);; South China Agricultural University, Guangzhou, China (K. Luo);; US Department of Agriculture, Starkville (F.L. Cunningham, K. Hanson-Dorr);; US Department of Agriculture, Fort Collins, Colorado, USA (J. Baroch, M.W. Lutman, T.J. DeLiberto);; St. Jude Children’s Research Hospital, Memphis, Tennessee, USA (R. Webby)

**Keywords:** Orthomyxoviridae, influenza A virus, influenza D virus, swine influenza, serology, zoonoses, viruses, United States

## Abstract

Influenza D virus (IDV) has been identified in domestic cattle, swine, camelid, and small ruminant populations across North America, Europe, Asia, South America, and Africa. Our study investigated seroprevalence and transmissibility of IDV in feral swine. During 2012–2013, we evaluated feral swine populations in 4 US states; of 256 swine tested, 57 (19.1%) were IDV seropositive. Among 96 archived influenza A virus–seropositive feral swine samples collected from 16 US states during 2010–2013, 41 (42.7%) were IDV seropositive. Infection studies demonstrated that IDV-inoculated feral swine shed virus 3–5 days postinoculation and seroconverted at 21 days postinoculation; 50% of in-contact naive feral swine shed virus, seroconverted, or both. Immunohistochemical staining showed viral antigen within epithelial cells of the respiratory tract, including trachea, soft palate, and lungs. Our findings suggest that feral swine might serve an important role in the ecology of IDV.

Influenza D virus (IDV), first isolated in 2011 from a domestic pig with influenza-like symptoms, has genomic similarity to influenza C virus (ICV) (*1*). IDV has 7 genomic RNA segments like ICV but exhibits a broader cellular and host tropism than ICV (*1*), which might be attributable to IDV’s open receptor-binding cavity (*2*). Evidence suggests that IDV circulates in domestic animals, including swine, cattle, camelids, and small ruminants, throughout North America, Asia, Africa, and South America (*1*,*3*–*15*). Among these species, cattle are proposed to be the natural reservoir of IDV (*13*,*15*). Susceptibility and seroprevalence of IDV in domestic and wild animal species is largely unknown.

Swine were introduced into what is now the United States in the 15th century. Since that time, populations of free-ranging swine have spread to ≈40 states. These swine are escaped domestic animals, imported wild boar, or hybrids of the two, and they now number ≈5 million (Figure 1, panel A) (*16*–*19*). Feral swine transmit diseases that are swine-specific (feral and domestic) as well as diseases that can be transmitted to domestic species (cattle, sheep, goats, horses, and dogs) and wild mammals; some of the more important diseases include porcine circovirus-2, pseudorabies virus, *Brucella suis*, and influenza A viruses (IAVs) as well as vesicular diseases (*16*,*17*,*20*–*25*). Feral swine have been shown to have contact with domestic swine in transitional and commercial settings (*17*,*20*). Moreover, feral swine also frequently interact with free-ranging cattle near shared water sources (*16*). Of particular concern, feral swine populations are increasing and pose potential threats to domestic swine and human public health (*26*).

**Figure 1:**
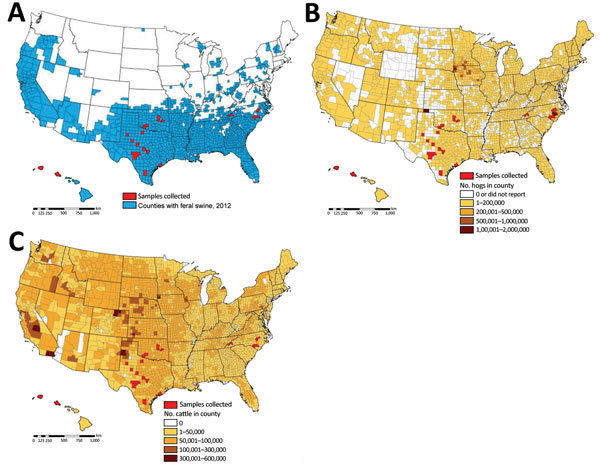
Geographic distribution of serum samples collected from feral swine (A), domestic swine (B), and domestic cattle (C), United States, October 1, 2012–September 30, 2013.

Little is known regarding seroprevalence of IDV in feral swine. In this study, we conducted serologic surveillance to estimate seroprevalence of IDV in the feral swine population in the United States. We also conducted infection experiments to determine the pathogenesis and transmission of IDV in feral swine.

## Materials and Methods

### Viruses

We used influenza viruses D/bovine/C00046N/Mississippi/2014 virus (D/46N) and D/bovine/C00013N/Mississippi/2014 virus (D/13N). Before use, the viruses were isolated and passaged twice in HRT-18G cells (American Type Culture Collection, Manassas, VA, USA) with Opti-MEM supplemented with 1× Pen Strep and 12.5× 7.5% bovine serum albumin (GIBCO Life Technologies, Carlsbad, CA, USA) and 1:2000 *N*-tosyl-L-phenylalanine chloromethyl ketone-Trypsin (Sigma-Aldrich, St. Louis, MO, USA).

### Serum Samples

A total of 256 convenient serum samples were available from a collection of feral swine serum archived by the US Department of Agriculture’s National Wildlife Research Center. The 256 samples constituted feral swine serum samples collected during October 1, 2012–September 30, 2013, in 4 US states: Hawaii (n = 73 samples), North Carolina (n = 64), Oklahoma (n = 49), and Texas (n = 70) (Table 1). North Carolina, Oklahoma, and Texas have large domestic swine populations; Texas and Oklahoma have large cattle populations; and Hawaii, Oklahoma, and Texas have large feral swine populations. Of the 256 serum samples, 118 were from male feral swine, 135 were from female feral swine, and 3 were from feral swine of unknown sex; in addition, 32 of the samples were from juveniles (<2 mo of age), 43 from subadults (2–12 mo of age), and 181 from adults (>1 y of age) (Table 2).

**Table 1:** Seroprevalence of influenza D virus among 256 feral swine, by state, United States, October 1, 2012–September 30, 2013*

State, no. samples	D/13N			D/46N		Total seropositive swine, no. (%)
	Seropositive swine, no. (%)	GMT (range)		Seropositive swine, no. (%)	GMT (range)	
Hawaii, n = 73	11 (16.4)	53.4 (1:40–1:80)		4 (5.5)	67.3 (1:40–1:80)	15 (20.5)
North Carolina, n = 64	4 (6.3)	67.3 (1:40–1:160)		3 (4.7)	40 (1:40–1:40)	5 (7.8)
Oklahoma, n = 49	13 (26.5)	49.5 (1:40–1:80)		3 (6.1)	50.4 (1:40–1:80)	14 (28.6)
Texas, n = 70	10 (14.3)	85.7 (1:40–1:160)		8 (11.4)	63.5 (1:40–1:160)	15 (21.4)

*D/13N and D/46N were used in HAI assays with 0.5% turkey red blood cells. Seropositivity defined as HAI titer >1:40. D/13N, influenza D/bovine/C00013N/Mississippi/2014 virus; D/46N, influenza D/bovine/C00046N/Mississippi/2014 virus; GMT, geometric mean titer; HAI, hemagglutination inhibition.

**Table 2:** Seroprevalence of influenza D virus among 256 feral swine, by age group and sex, Hawaii, North Carolina, Oklahoma, and Texas, United States, October 1, 2012–September 30, 2013*

Characteristic, no. samples	D/13N			D/46N		Total seropositive swine, no. (%)
	Seropositive swine, no. (%)	GMT (range)		Seropositive swine, no. (%)	GMT (range)	
Age						
Juvenile, n = 32	6 (18.8)	63.5 (1:40–1:160)		1 (3.1)	80.0 (1:80–1:80)	7 (21.9)
Subadult, n = 43	6 (14.0)	40.0 (1:40–1:40)		2 (4.7)	40.0 (1:40–1:40)	8 (18.6)
Adult, n = 181	27 (14.9)	65.1 (1:40–1:160)		15 (8.3)	58.8 (1:40–1:160)	34 (18.8)
Sex						
F, n = 135	23 (17.0)	62.9 (1:40–1:160)		10 (7.4)	54.0 (1:40–1:160)	28 (20.7)
M, n = 118	16 (13.6)	56.6 (1:40–1:160)		8 (6.8)	61. 7 (1:40–1:160)	21 (17.8)

*D/13N and D/46N were used in HAI assays with 0.5% turkey red blood cells. Seropositivity defined as HAI titer >1:40. Sex unknown for 3 animals. D/13N, influenza D/bovine/C00013N/Mississippi/2014 virus; D/46N, influenza D/bovine/C00046N/Mississippi/2014 virus; GMT, geometric mean titer; HAI, hemagglutination inhibition.

Previous studies have suggested that feral swine have been exposed to IAV (*24*,*25*). To determine whether feral swine could have been exposed to both IDV and IAV, we identified 96 IAV seropositive samples. Of the total 256 samples we described, 13 were IAV-seropositive and were included in the IAV-seropositive sample set. We selected 83 additional convenient serum samples collected during October 1, 2012–September 30, 2013, from archived feral swine serum samples that had been previously determined to be IAV seropositive using the IDEXX AI MultiS-Screen Ab Test (IDEXX, Westbrook, ME, USA) (*9*,*10*). We sampled these 96 IAV-seropositive samples from 16 US states: Alabama (n = 7 samples), Arizona (n = 1), California (n = 5), Florida (n = 6), Georgia (n = 8), Hawaii (n = 4), Illinois (n = 2), Kansas (n = 5), Louisiana (n = 2), Missouri (n = 1), North Carolina (n = 8), New Mexico (n = 1), Oklahoma (n = 9), South Carolina (n = 1), Tennessee (n = 2), and Texas (n = 34).

### Hemagglutination Assays

We performed hemagglutination (HA) and HA inhibition (HAI) assays as previously described (*11*). In brief, we treated serum samples with receptor-destroying enzyme (Denka Seiken Co., Tokyo, Japan) at 37°C for >18 h, followed by heat inactivation at 55°C for 30 min. We diluted inactivated serum to a final concentration of 1:10 with 1× phosphate-buffered saline. We added turkey red blood cells to the serum (concentration 1:20) at 4°C for 30 min and then centrifuged the serum at 13,000 rpm for 1 min to pellet the red blood cells. We conducted the HA and HAI assays with 0.5% turkey red blood cells at 4°C against a testing IDV; we considered samples with an HAI titer >1:40 as IDV seropositive. We tested all serum samples against influenza D/46N and D/13N.

### Infection Experiments

We trapped a total of 26 feral swine over the course of 100 trap nights in Mississippi and transported them to the US Department of Agriculture’s Mississippi Field Station under state permits (nos. 894, 896, and 908). All 26 feral swine tested seronegative for pseudorabies, brucellosis, and IDVs (D/13N and D/46N).

We randomly separated the 26 feral swine into 3 groups: virus-inoculated animals (n = 12), contact animals (n = 8), and control animals (n = 6). We used 12 pens to house the 26 feral swine; 4 pens contained 1 virus-inoculated animal plus 1 contact animal, 4 pens contained 2 virus-inoculated animals plus 1 contact animal, and 3 pens contained 2 control animals. Pens housing control feral swine were in an animal room separate from pens housing contact and inoculated feral swine. We inoculated each animal for the virus-inoculated group intranasally with 1 mL of D/46N (10^6^ 50% tissue culture infective dose [TCID_50_]) and each control animal with 1 mL of phosphate-buffered saline. At 2 days postinoculation (dpi), we moved 1 contact animal into the pen housing 1 or 2 virus-inoculated feral swine. At 0, 3, 5, 7, 9, 11, and 21 dpi, we collected nasal washes, rectal swab samples, and blood from all animals. We stored nasal washes and rectal swab samples at −80°C and serum samples at −20°C. 

We euthanized feral swine at 3 dpi (1 virus-inoculated, 1 contact, and 1 control animal), 5 dpi (3 virus-inoculated, 1 contact, and 2 control animals), 7 dpi (3 virus-inoculated, 1 contact, and 1 control animal), 9 dpi (2 virus-inoculated, 1 contact, and 1 control animal), 11 dpi (2 virus-inoculated and 1 contact animal), and 21 dpi (1 virus-inoculated, 3 contact, and 1 control animal). We collected nasal turbinate, soft palate, trachea, bronchi, and lung at necropsy and stored tissues at −80°C before virologic characterization or fixed tissues in 10% neutral buffered formalin for histologic and immunohistochemical analysis.

### Calculation of TCID_50_

We serially diluted homogenate supernatants from tissue samples and nasal washes from 10^−1^ to 10^−6^ and titrated them in HRT-18G cells. We tested each sample in triplicate and calculated the TCID_50_ by using the Reed-Muench method (*27*).

### Immunohistochemical Staining

We performed IDV immunohistochemical staining as previously described (*28*). In brief, we heated slides at 65°C overnight. We deparaffinized and retrieved slides in an antigen retrieval solution at pH 6.1 (Dako, Carpinteria, CA, USA) by using a decloaking chamber. We used Tris-buffered saline (TBS) with 0.5% Tween to wash slides and used 3% H_2_O_2_ to quench peroxidase activity; we then blocked slides with 10% normal goat serum (Invitrogen, Carlsbad, CA, USA) for 1 h. We added bovine-generated antiserum to D/46N (diluted 1:200 in antibody diluent [Dako]) to the slides and incubated them at −4°C for 24 h. We then washed the slides with TBS with 0.5% Tween and incubated them for 30 min with biotinylated goat anti–bovine IgG polyclonal secondary antibody diluted 1:500 in TBS with 0.5% Tween. Slides were washed and then incubated with ABC reagent (Vectastain, Burlingame, CA) according to the manufacturer’s instructions, exposed to 3,3′-diaminobenzidine and H_2_O_2_ for 5 min, counterstained with hematoxylin, and dehydrated; we then applied a coverslip.

### Biosafety and Animal Handling

We conducted laboratory and animal experiments under Biosafety Level 2 conditions in compliance with protocols approved (QA 2563) by the Institutional Animal Care and Use Committee of the US Department of Agriculture’s National Wildlife Research Center. Before necropsy, we fully anesthetized the feral swine with 0.044 mL/kg TKX (Telazol 4.4 mg/kg, ketamine 2.2 mg/kg, and xylazine 2.2 mg/kg) and, once fully sedated, the swine were euthanized by administration of a barbiturate solution (1 mL/4.5 kg body weight).

## Results

To identify whether IDV is circulating among feral swine populations in the United States, we performed HAI against D/46N and D/13N on 256 serum samples from feral swine collected during October 1, 2012–September 30, 2013 (*11*). We selected D/46N and D/13N to represent 2 genetic clades of IDVs, which are antigenically different (*13*,*29*). Of the 256 samples, 39 (15%) were positive for D/13N (HAI geometric mean titer [GMT] 60.2, range 1:40–1:160), and 18 (7%) were positive for D/46N (HAI GMT 52.3, range 1:40–1:160); the overall seropositive rate was 19.1% for IDV. Of the 39 samples seropositive for D/13N, 8 were also seropositive for D/46N, but the other 31 samples were seronegative for D/46N; of the 18 samples seropositive for D/46N, 10 samples were also seropositive for D/13N, whereas the other 8 samples were seronegative for D/13N. These data suggest a greater prevalence of infection with viruses antigenically related to D/13N among the feral swine populations tested.

The overall seroprevalence rate for IDV (i.e., D/13N, D/46N, or both) was 21.9% in juveniles (n = 32), 18.6% in subadults (n = 43), and 18.8% in adults (n = 181) (Table 2). Overall, seroprevalence was 17.8% among female feral swine (n = 135) and 20.7% among male feral swine (n = 118) (Table 2). The sex of 3 feral swine was unknown, but the animals were seronegative for IDV. By state, IDV seropositive rates among feral swine were 20.5% in Hawaii, 7.8% in North Carolina, 28.6% in Oklahoma, and 21.4% in Texas (Table 1; Technical Appendix).

Previous serologic surveillance showed that ≈4.9% of feral swine had been exposed to IAVs (*25*). We explored whether an opportunity exists for feral swine to be exposed to both IAV and IDV. Our results show that 13 (5.1%) of the 256 serum samples were IAV positive (Table 1, 2), and 5 (38.5%) of the 13 were IDV positive. To determine whether feral swine could have exposure to both IAV and IDV, we selected an additional 83 serum samples from the 294 IAV-positive samples collected during October 1, 2012–September 30, 2013. We tested the 96 IAV feral swine serum samples (including the 13 already discussed) against D/46N and D/13N; of the 96 samples, 41 (42.7%) were IDV seropositive for D/13N (n = 37; GMT 1:59.6, range 1:40–1:160), D/46N (n = 9; GMT 1:58.8, range 1:40–1:160), or both (n = 5; GMT1:59.3, range 1:40–1:160) (Technical Appendix Table 2).

**Figure 2:**
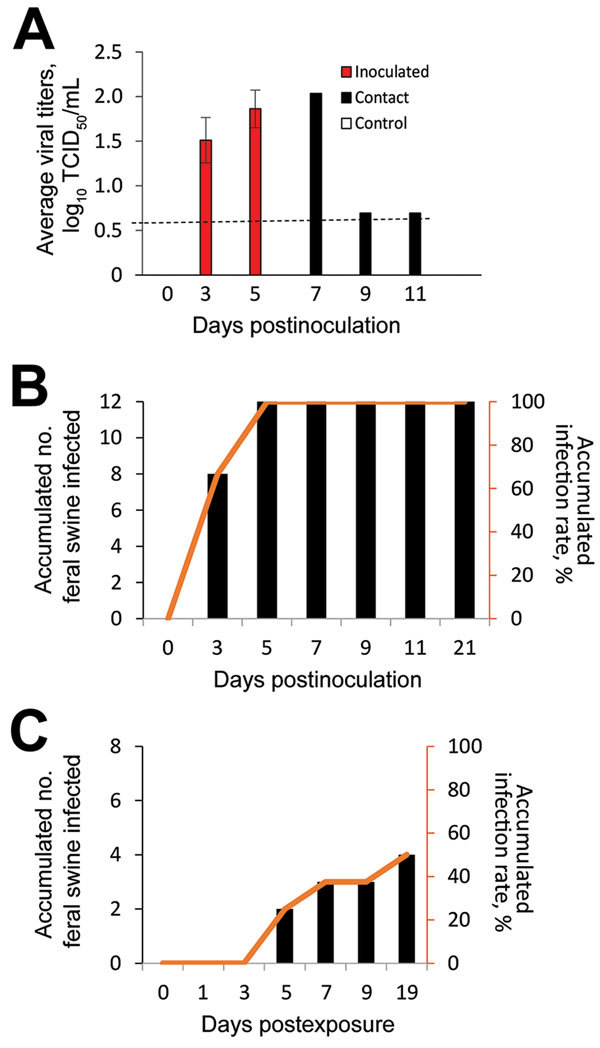
Infectivity and transmissibility of influenza D virus in feral swine populations, United States. A) Viral titers from nasal washes of feral swine. Feral swine were inoculated intranasally with 10^6^ TCID_50_/mL of influenza D/bovine/C00046N/Mississippi/2014 virus. Naive feral swine were exposed to the virus by direct contact with D/bovine/C00046N/Mississippi/2014 virus–inoculated feral swine. On days 3, 5, 7, 9, and 11 after the inoculation group was inoculated, nasal washes were collected from all 3 groups of swine and in HRT-18G cells. Ending titers are expressed as log_10_ TCID_50_/mL. The limit of virus detection was 10^0.699^ TCID_50_/mL. Error bars represent standard error of viral titers. The dashed line indicates the lower limit of detection, which is 10^0.699^ TCID_50_/mL. B) Accumulated number of feral swine infected and accumulated infection rate for the feral swine inoculated with influenza D virus. C) Accumulated number of feral swine infected and accumulated infection rate for the contact feral swine. A feral swine was considered infected if a viral titer was detected in in nasal washes, serum samples, or both or if this feral swine seroconverted. TCID_50_, 50% tissue culture infective dose.

To evaluate the characteristics of IDV infection in feral swine, we inoculated D/46N virus intranasally into 12 feral swine. We chose D/46N because this virus was shown to cause infection and transmission as well as a substantial increase in neutrophil tracking in tracheal epithelia of the infected calves, and we intended to compare the pathogenesis in cattle with that in feral swine (*28*). The IDV-inoculated swine showed no clinical signs or changes in body temperature. Viral titration of nasal washes showed that, at 3 dpi, 7 of 12 D/46N-inoculated swine shed virus with a maximum titer of 2.199 log_10_ TCID_50_/mL, and that, at 5 dpi, 6 of the 8 remaining virus-inoculated swine shed virus with a maximum titer of 2.366 log_10_ TCID_50_/mL. None of the remaining 5 virus-inoculated swine shed virus at or after 7 dpi (Figure 2; Technical Appendix Table 3). No virus was detected in any rectal swab samples from these experimentally infected feral swine.

HAI results indicated that 7 (63.6%) of 11 virus-inoculated animals seroconverted at 5 dpi and all 8 remaining virus-inoculated animals seroconverted at 7 dpi (Technical Appendix Table 4). We did not detect virus in any fecal swab samples from virus-inoculated swine, nor did we detect virus in nasal washes or fecal swab samples from the control feral swine, which remained seronegative throughout the study.

The viral titrations of feral swine tissues demonstrated viral replication in the upper and lower respiratory tract as well as the soft palate. At 5 dpi, viral titers were highest in the trachea sections (2.699–2.366 log_10_ TCID_50_/mL) and lowest in the left and right caudal lung and soft palate (0.699–2.199 log_10_ TCID_50_/mL _0_) (Technical Appendix Table 3). At 7 dpi, we detected no virus in nasal swab samples; however, the highest (maximum) viral titer (3.866 log_10_ TCID_50_/mL) was in the soft palate, and the lowest viral titers (0.699 log_10_ TCID_50_/mL) in the lower trachea (Technical Appendix Table 3).

Three of the 8 contact animals exposed to IDV infected feral swine had detectable viral shedding: 1 animal shed virus at 5 days postexposure (titer 2.032 log_10_ TCID_50_/mL), and 1 animal each shed virus at 7 and 9 days postexposure (both had a titer equal to the detection limit). At 19 days postexposure, 1 of the 3 remaining contact feral swine seroconverted, with an HAI titer of 1:40, indicating that IDV can be transmitted among feral swine (Table 3; Technical Appendix Table 4). Nearly half of the contact animals exposed to IDV-infected feral swine were infected with IDV (Figure 2). We did not detect virus in any rectal swab samples from the contact feral swine. None of the contact animals showed any clinical signs or change in body temperature.

**Table 3:** Summary of viral shedding and seroconversion in evaluation of characteristics of influenza D virus infection in feral swine*

Timeline	HAI titer	Nasal titer
Inoculated swine, dpi, n = 12		
3	0 (12)	7 (12)
5	6 (11)	6 (11)
7	8 (8)	0 (8)
9	5 (5)	0 (5)
11	3 (3)	0 (3)
21	1 (1)	0 (1)
Control swine, dpi, n = 6‡		
3	0 (6)	0 (6)
5	0 (5)	0 (5)
7	0 (3)	0 (3)
9	0 (2)	0 (2)
11	0 (1)	0 (1)
21	0 (1)	0 (1)
Contact swine, dpe, n = 8§		
1	0 (8)	0 (8)
3	0 (7)	0 (7)
5	0 (6)	1 (6)
7	0 (5)	1 (5)
9	0 (4)	1 (4)
19	1 (3)	0 (3)

*HAI titer data indicate number of swine that seroconverted (no. tested). Seropositivity defined as HAI titer >1:40. Nasal titer data indicate number of swine that shed virus (no. tested). Animals with a nasal wash viral titer >0.699 log_10_ TCID_50_/mL were considered as shedding virus. dpe, days postexposure; dpi, days postinoculation; HAI, hemagglutination inhibition; TCID_50_, 50% tissue culture infective dose. †Feral swine inoculated with influenza D/bovine/C00046N/Mississippi/2014 virus. ‡Feral swine inoculated with sterile phosphate-buffered saline. §Feral swine with direct-contact exposure to influenza D virus–inoculated swine.

IDV causes viremia in IDV-inoculated feral swine and in feral swine that have direct contact with infected animals. Among the IDV-inoculated animals, we detected viremia in 3 animals (nos. 103, 105, and 125) at 3 dpi (titer 3.199 log_10_ TCID_50_/mL) and in 1 animal (no. 125) at 5 dpi (titer 2.199 log_10_ TCID_50_/mL). Among the contact animals, we detected viremia in 1 animal (no. 118) at 7 days postexposure (titer 2.199 log_10_ TCID_50_/mL) (Technical Appendix Table 4).

**Figure 3:**
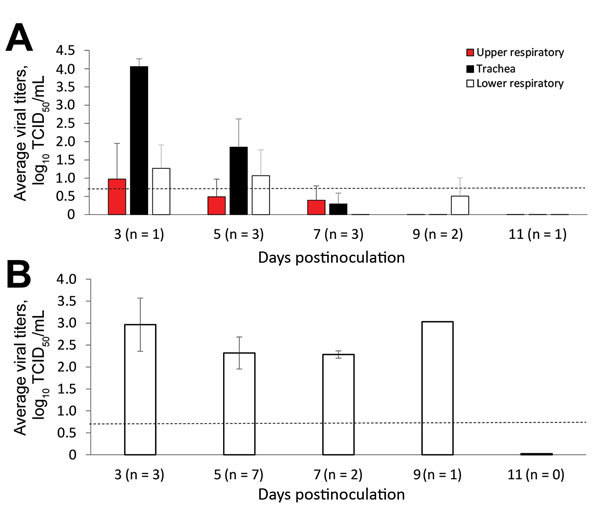
Influenza D viral titers in feral swine tissues. Feral swine were inoculated intranasally with 10^6^ TCID_50_ of influenza D/bovine/C00046N/Mississippi/2014 virus or sterile phosphate-buffered saline (controls). At 3, 5, 7, 9, and 11 days postinoculation, they were humanely euthanized, and the following tissues were collected: turbinate; soft palate; upper, middle, and lower trachea; bronchus; left and right caudal lung, left and right medial lung, left and right cranial lung; and right accessory lung. A) The tissues were grouped as upper respiratory tract (turbinate and soft palate), trachea (upper, middle, and lower trachea, and bronchus), and lower respiratory tract (left and right caudal lung; left and right medial lung; and left and right cranial lung). B) All lung tissue sections at each time point that were influenza D virus–positive by TCID_50_ titration in HRT-18G cells were averaged and plotted for each day postinoculation; day 9 has no error bars because only 1 positive tissue sample was found. Dashed lines indicate the lower limit of detection, which was 10^0.699^ TCID_50_/mL. Error bars indicate SE. Numbers in parentheses indicate number of animals used in the analyses. TCID_50_, 50% tissue culture infective dose.

Viral titration showed that virus was in the nasal turbinate, soft palate, trachea, lung tissues, or some combination of these tissues collected at 3–9 dpi. The tissue with the highest viral titer (5.366 log_10_ TCID_50_/mL) was the lower trachea of an IDV-inoculated animal at 5 dpi. Immunohistochemical staining demonstrated the presence of IDV antigen in epithelial cells of the soft palate, trachea, and lung. In the lung, we observed IDV immunostaining in type I pneumocytes, macrophages, and bronchiolar epithelial cells (Figure 3). The viral titrations on tissues from contact animals indicated that they were negative for IDV.

## Discussion

Since it was first identified in domestic swine in 2011, IDV has been detected in a wide range of animal hosts, mainly livestock such as bovids, goats, horses, and sheep, across multiple continents, indicating that IDV is a transboundary pathogen (*12*–*19*,*30*). In the United States, feral swine serve as an important vector between domestic and wild animals for multiple transboundary diseases, such as *Brucella suis* and IAV (*20*–*25*). Our study demonstrated that the seroprevalence rate of IDV in feral swine was 19.1%, which is similar to rates (13.5%–18.3%) reported for commercial beef cattle (*15*) but higher than the reported rate (<10%) in domestic swine (*1*). For example, a serologic study that used 220 pigs (3–20 weeks old) found that only 9.5% of tested domestic swine had an IDV titer ≥1:10, with a GMT of 1:20.7 (*1*). The difference in the seroprevalence between domestic and feral swine might be related to the fact that feral swine are mobile and have ample opportunities to come into contact with various domestic and wild animals; thus, compared with domestic swine, feral swine could have additional opportunities to be exposed to IDV. Among the 4 states that we sampled, the state with the highest IDV seroprevalence rate in feral swine also has the largest cattle population (Figure 1); however, whether IDV transmissions between bovids and feral swine is bidirectional is unknown. Two previous studies suggested that feral swine are likely to have indirect and direct contact with free-range bovine herds near water sources and that higher *B. suis* seroprevalence among free-range bovine herds was likely attributable to the bovine herd’s close proximity to feral swine (*16*,*23*). Nevertheless, the seroprevalence rate reported in our study among feral swine was based on convenience samples; thus, an epidemiologic study will be needed to determine the enzootic status of IDV in the feral swine population.

Previous studies have suggested that domestic swine are major sources of IAV exposure for feral swine (*24*,*25*). IDV was shown not to cross-react with IAV (*1*), and our study showed that 42.3% of the IAV-seropositive feral swine had exposure to IDV, indicating that feral swine were exposed to both IDV and IAV. In addition, our results showed that the seroprevalence rate of IDV in IAV-seropositive feral swine was more than twice that observed among IAV-negative feral swine. However, a larger epidemiologic study covering larger geographic areas and longer periods is needed to test the hypothesis that IDV would be more prevalent in IAV-positive than IAV-negative feral swine. The effect of IDV infections on the pathogenesis of IAV, or vice versa, remains unknown, but findings from our study and previous studies suggest that future work ought to focus on whether feral swine act as a vector for transboundary disease between domestic swine and cattle.

**Figure 4:**
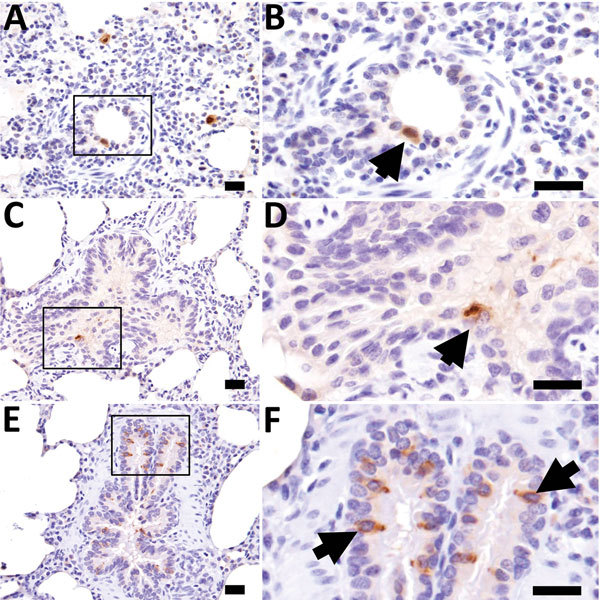
Influenza D virus immunohistochemistry in swine lung at 3 days (A and B), 5 days (C and D), and 7 days (E and F) postinoculation. Right column panels are higher magnification of boxed region in panels to the left. At all time points, scattered immunopositive bronchiolar epithelial cells were observed (arrows). Scale bars indicate 20 µm.

The results of our animal challenge study show that IDV can be transmitted among feral swine; however, the infection resulted in limited clinical signs. Cattle infected with IDV shed virus up to 9 dpi, with a peak titer of 4.417 log_10_ TCID_50_/mL (*28*), whereas the swine infected with IDV in our study shed virus only up to 5 dpi, with a peak titer of 2.366 log_10_ TCID_50_/mL (Figure 2). Such a difference might be attributable to the distinct patterns of viral distributions in the respiratory tracts of the infected animals. In cattle, IDV was predominantly distributed in the tissues of the upper respiratory tract (i.e., turbinate and trachea) (*28*), but in swine the distribution was predominantly in the middle and lower respiratory tract (i.e., trachea and lung) (Figure 4, panel A). Previous studies have suggested that, under laboratory conditions, IDV replicates in the upper and lower respiratory tracts of guinea pigs infected with a bovine strain of IDV (*6*). In addition, bovine IDV infects ferrets and can be transmitted to IDV-naive ferrets through direct contact; however, IDV cannot infect naive ferrets through a fomite contaminated with nasal drainage from IDV-infected calves (*1*,*28*). Another swine infection study indicated that no viruses were detected in the lung of domestic swine that were infected with D/swine/Oklahoma/1334/2011 (*1*). The genetic variations between D/swine/Oklahoma/1334/2011and D/46N used in these studies might have led to the difference in viral tissue tropisms observed in these 2 studies.

Three IDV-inoculated animals and 1 contact animal in our study had transient viremia, a finding consistent with a previous study that found IDV in animal serum samples during IDV surveillance (*31*). We found IDV at moderate viral titers in the soft palate of feral swine at 3 dpi, and the viremia lasted >3 days for some animals (Technical Appendix Table 3). The soft palate has been identified as a major site of influenza virus infection in ferrets (*32*). Previous studies have shown that several bacteria (e.g., *Streptococcus porcinus, Streptococcus dysgalactiae, Staphylococcus aureus, Staphylococcus hyicus, Streptococcus suis, Yersinia enterocolitica, Salmonella* spp., and *Listeria monocytogenes*) and viruses (e.g., porcine reproductive and respiratory syndrome virus and porcine circovirus-2) can be isolated from the soft palate (*33*). The rich distribution of lymphoid tissue in the soft palate might enable virus from the soft palate to enter the bloodstream and cause the transient viremia observed in feral swine that we observed in the experimentally infected feral swine.

The transmission ability of IDV through direct contact is similar to that in domestic swine; however, the transmission ability in feral and domestic swine seems to be less efficient than that in bovids, as suggested by viral load titers and durations of shedding (*1*,*28*). Given the limited transmissibility of IDV in feral swine, we would speculate that feral swine could have additional opportunities for exposure to IDV in addition to IDVs circulating in the feral swine populations.

In summary, our findings suggest that IDV has been circulating in the feral swine population across multiple states in the United States and that IDV can be transmitted among feral swine. Although the economic impact of IDV on commercial livestock remains unknown, our findings suggest that feral swine might be important in the ecology of IDV. Further studies are needed to understand whether other wild animals are infected by IDV and to what extent interspecies transmission contributes to IDV maintenance in domestic and wild populations.

Technical Appendix Supplementary information on serologic and virologic data for influenza D virus exposures or infections in feral swine populations, United States.
